# Mechanisms of Shared Vulnerability to Post-traumatic Stress Disorder and Substance Use Disorders

**DOI:** 10.3389/fnbeh.2020.00006

**Published:** 2020-01-31

**Authors:** Cristina E. María-Ríos, Jonathan D. Morrow

**Affiliations:** ^1^Neuroscience Graduate Program, University of Michigan, Ann Arbor, MI, United States; ^2^Department of Psychiatry, University of Michigan, Ann Arbor, MI, United States

**Keywords:** comorbidity, self-medication, sensitization, individual differences, dual-diagnosis

## Abstract

Psychoactive substance use is a nearly universal human behavior, but a significant minority of people who use addictive substances will go on to develop an addictive disorder. Similarly, though ~90% of people experience traumatic events in their lifetime, only ~10% ever develop post-traumatic stress disorder (PTSD). Substance use disorders (SUD) and PTSD are highly comorbid, occurring in the same individual far more often than would be predicted by chance given the respective prevalence of each disorder. Some possible reasons that have been proposed for the relationship between PTSD and SUD are self-medication of anxiety with drugs or alcohol, increased exposure to traumatic events due to activities involved in acquiring illegal substances, or addictive substances altering the brain’s stress response systems to make users more vulnerable to PTSD. Yet another possibility is that some people have an intrinsic vulnerability that predisposes them to both PTSD and SUD. In this review, we integrate clinical and animal data to explore these possible etiological links between SUD and PTSD, with an emphasis on interactions between dopaminergic, adrenocorticotropic, GABAergic, and glutamatergic neurobehavioral mechanisms that underlie different emotional learning styles.

## Introduction

Most people will experience a traumatic event in their lifetime. It is normal to exhibit fear during a traumatic situation and to have strong reactions afterward, such as flashbacks and nightmares. Perceived threats induce stereotyped reactions in the mind and body that are meant to cause individuals to respond appropriately and protect themselves from harmful situations. Even though these fear reactions during and after the traumatic experience are not unusual, it is vital that they subside with time. Out of the nearly 90% of adults in the United States that experience a traumatic event, about 10% cannot recover naturally from the trauma and continue to feel in danger and exhibit high levels of stress even when they are not in a dangerous situation (Kilpatrick et al., 2013). This persistent fear is characteristic of post-traumatic stress disorder (PTSD), a debilitating neuropsychiatric illness that causes individuals to continually suffer from emotional distress even years after experiencing the trauma. While PTSD and substance use disorders (SUD) are phenomenologically distinct in many obvious ways, this review will highlight similar neuropsychiatric processes that can lead to the pathologically intense emotional and motivational reactions that characterize both these disorders.

PTSD most commonly presents in people who have experienced natural disasters, terrorist attacks, war, violent and sexual assaults, and other life-threatening incidents (Kessler et al., 1995; Creamer et al., 2001). Both women and men can develop PTSD, but it is twice as common in women (Dell’Osso et al., 2011). The first studies on PTSD came mostly from male war veterans (especially Vietnam), but with time researchers started noticing that women who experienced sexual assault showed very similar symptoms to male veterans (Kardiner, 1941; Figley, 1978; American Psychiatric Association, 1980; Herman, 1997). This led to increased interest in studying PTSD in both males and females, and to expanding the categories of traumatic experiences considered capable of causing PTSD (Lasiuk and Hegadoren, 2006). As it is currently defined, patients with PTSD must fit several criteria. The person should have experienced a traumatic event (Criterion A) and must be experiencing symptoms in each of four different clusters. The first cluster (Criterion B) is experiencing intrusive memories or re-experiencing the traumatic event, including nightmares, flashbacks, and both psychological and physiological reactions to reminders of the event. The second set of symptoms (Criterion C) are of avoidance, which includes avoiding the thoughts and feelings associated with the event as well as the people tied to it. The third group of symptoms (Criterion D) is negative alterations in mood and cognition, which encompass memory problems exclusive to the event, negative thoughts and sense of blame for one’s self and others, reduced interest in engaging in activities, and detachment and isolation from other people. The last set of symptoms (Criterion E) are increased arousal, described as irritability and anger, hypervigilance, difficulty sleeping and, in general, feeling “on edge” (American Psychiatric Association, 2013).

Epidemiological evidence suggests a close relationship between PTSD and SUD. As many as 50–75% of combat veterans with PTSD also have drug or alcohol use disorders (Kulka et al., 1990), and structured interviews detect PTSD in up to 42.5% of patients in inpatient substance abuse programs (Cottler et al., 1992). As devastating as PTSD can be, its clinical course often seems to be worsened by its relationship with SUD. Studies have consistently shown that the co-occurrence of PTSD and SUD makes each individual condition more severe and difficult to treat (Saladin et al., 1995; Ouimette et al., 1996, 1998; Clark et al., 2001). Patients with comorbid PTSD and SUD have poorer mental health functioning, poorer treatment adherence and response, more inpatient hospitalizations, worse physical health, and more interpersonal problems (Brown et al., 1995; Stevens et al., 2003; Ouimette et al., 2006; Norman et al., 2007; Driessen et al., 2008). Patients tend to believe their own PTSD and SUD are functionally related and prefer concurrent, integrated treatment (Brown et al., 1998). Clinicians view these dual-diagnosis patients as particularly challenging, in part because they feel uncertain how best to prioritize and integrate treatment of the two disorders (Najavits, 2002; Back et al., 2009).

**Figure 1:**
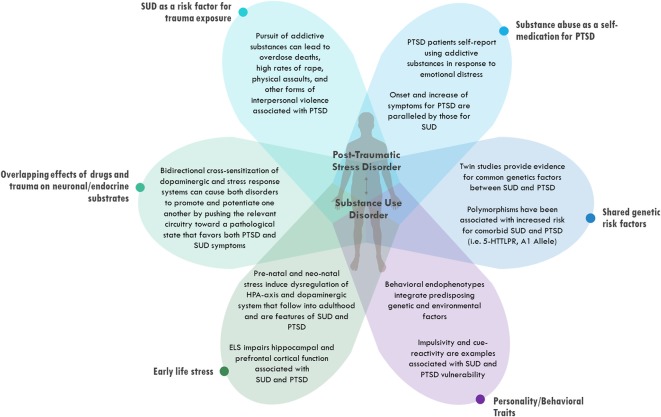
Possible etiologies for comorbid post-traumatic stress disorder (PTSD) and substance use disorders (SUD). Different categories of explanations are depicted as being distinct from one another conceptually but overlapping at the level of the individual patient.

In this review article, data from both clinical populations and animal models are presented to highlight the high prevalence of PTSD and SUD comorbidity and propose possible etiological factors that might explain their co-occurrence. Many possible explanations have been proposed for the relationship between PTSD and SUD, and several of these will be considered in turn (Figure 1). First, evidence is presented suggesting that the negative consequences of seeking and using addictive drugs may increase exposure to traumatic events, thereby raising the risk of developing PTSD. An analogous idea is then explored that PTSD may increase exposure to addictive drugs through attempts to self-medicate psychiatric symptoms with drugs or alcohol. Next, some overlapping mechanisms of trauma and abuse substances that alter neural and endocrine signals and increase vulnerability to both PTSD and SUD are highlighted. Finally, the focus of the review turns toward intrinsic vulnerability factors that may predispose certain individuals to both PTSD and SUD, including both genetic factors and early life events. The potential role of different emotional learning styles in predisposing some individuals to develop neuropsychiatric disorders is also explored. It is important to point out that these different explanations are not mutually exclusive, and there is evidence to support each of them. Most cases of comorbid PTSD and SUD are likely due to a combination of several of these processes acting simultaneously on the same individual.

## Substance Use as a Risk Factor for Trauma Exposure

The first possibility to consider is that SUD in effect causes PTSD by exposing the individual to traumatic stressors resulting from the pursuit and use of addictive substances. One commonality between SUD and PTSD is their incontrovertible dependence on instigating environmental factors. Just as the development of SUD requires exposure to addictive substances, PTSD requires exposure to traumatic events (American Psychiatric Association, 2013). Patients with SUD necessarily are involved in risky substance use, and this kind of use can substantially increase the chances of encountering a wide range of traumatic experiences. Overdose deaths are common in this population, and high rates of rape, physical assaults, and other forms of interpersonal violence have been documented as well (Clark et al., 2001; Johnson et al., 2003, 2006; Kingston and Raghavan, 2009; Lee et al., 2018). One study found that almost a third of traumas resulting in PTSD among SUD patients occurred as a direct result of the use or procurement of illicit substances (Brady et al., 1998). Data from the St. Louis Catchment Area study indicated that users of cocaine and opioids were more than three times as likely as the general population to report a history of trauma, most commonly interpersonal violence (Cottler et al., 1992). An earlier cross-sectional population study of 3,132 adults suggested a more complex relationship between substance use and trauma, with a history of sexual assault emerging as a risk factor for the development of SUD, and then the SUD, in turn, was a risk factor for subsequent assaults (Burnam et al., 1988). As we will see, however, this is but one of several possible connections between PTSD and SUD.

## Substance Abuse as Self-medication for PTSD Symptoms

Perhaps the most widely accepted explanation for the relationship between PTSD and SUD is the self-medication hypothesis which essentially posits that high rates of SUD are the result of patients using addictive substances to self-medicate their PTSD symptoms (Khantzian, 1985, 1997). For example, a longitudinal study in 1996 on 61 Vietnam veterans investigated the course of illness for both PTSD and SUD symptoms and reported the effects of abused substances on the symptoms of PTSD. They found that most patients developed symptoms like re-experiencing, hyperarousal, and avoidance within 2 years of exposure to combat, with a smaller percentage developing them during the combat tour, and others not meeting full PTSD criteria until 10 years after the combat. Interestingly, they found that the course of alcohol and substance abuse followed the same pattern as the PTSD symptoms. In comparison to 2 years before the war, there was a significant increase at every time point evaluated after the war for the use of alcohol, heroin, cocaine, and marijuana that lasted up to 24 years after the trauma. Overall, these findings suggest that the onset and increase of symptoms for PTSD are closely paralleled by those for alcohol and substance abuse. Additionally, most patients reported that the use of alcohol, marijuana, benzodiazepines, and heroin reduced their PTSD symptoms, supporting the hypothesis that patients are using these substances in order to self-medicate (Bremner et al., 1996). Similar studies in both military and civilian populations have identified PTSD as a prospective risk factor for SUD, and have found that PTSD patients self-report using addictive substances in response to emotional distress (Shipherd et al., 2005; Ullman et al., 2005; Reed et al., 2007; Waldrop et al., 2007; Haller and Chassin, 2014; McDevitt-Murphy et al., 2015).

One important clinical implication of the self-medication hypothesis is that, because PTSD symptoms are the primary drivers of substance use in these patients, effective treatment for comorbid PTSD and SUD should focus primarily on the PTSD symptoms. A “sequential treatment” strategy resulting first in significant improvement of PTSD symptoms should subsequently reduce the need for self-medication and lead to an improvement in SUD outcomes that would otherwise be difficult to achieve. Several clinical trials have attempted to identify effective strategies for the treatment of comorbid PTSD and SUD, but as noted in a recent Cochrane review, these experiments have generally been plagued by high attrition rates and suboptimal study designs (Roberts et al., 2015). Exposure therapy is a highly effective treatment for PTSD involving exposure to trauma-related stimuli that is continued until the fear/anxiety response subsides (Foa et al., 2005, 2008; Wood et al., 2009; Cusack et al., 2016). While several studies have found exposure therapy to improve PTSD outcomes in patients with comorbid PTSD and SUD, none has reported significant improvements in SUD outcomes relative to controls (Simpson et al., 2017). Few studies have focused on the effects of SUD-specific treatments on PTSD symptoms in this population. One Australian randomized controlled trial tested the efficacy of the Flinders Program of SUD-focused care management, which includes individualized delivery of self-management skills, medication adherence strategies, motivational enhancement, problem-solving, and health-care system navigation, for a sample of 77 Vietnam veterans with alcohol use disorder, almost all of whom also had comorbid PTSD. The group that received the Flinders Program intervention showed greater improvement in SUD outcomes than controls, but there were no group differences in PTSD outcomes (Battersby et al., 2013). The preponderance of evidence suggests that integrated treatments designed to address both SUD and PTSD simultaneously may be associated with better outcomes than sequential treatment (McGovern et al., 2011, 2015; Boden et al., 2012). Though the potential clinical utility of this information is clear, it does little to shed light on the etiological links between SUD and PTSD.

Patients with PTSD often cite their psychiatric symptoms as the reason they use addictive drugs. However, a strong argument can be made that if a patient truly is primarily using substances as part of a conscious strategy to reduce his or her PTSD symptoms, that person does not really have an SUD. SUDs fundamentally involve a loss of control over substance use such that conscious plans, strategies, and explanations for substance use no longer match up with the behavior [National Institute on Alcohol Abuse and Alcoholism (NIAAA) (2019); National Institute on Drug Abuse (NIDA) (2019)]. This does not negate the potential importance of self-medication in the etiology of comorbid PTSD and SUD; what starts out as self-treatment of PTSD symptoms can expose individuals to high levels of substance use, thereby greatly increasing their risk of developing a SUD. However, as the addictive process takes hold the substance use will gradually begin to take on a life of its own and may, therefore, be expected to continue even after the instigating psychiatric symptoms are under good control.

A somewhat different version of the self-medication hypothesis was proposed by Volpicelli et al. (1999). The model went beyond the initial concept that during times of stress, alcohol is used to reduce anxiety levels. Based on the observation that rats tend to increase their alcohol preference days after the stress and not during the days of stress exposure (Volpicelli et al., 1990), they hypothesize that the increase in alcohol consumption seen after a traumatic experience—like that observed on PTSD patients—is more related to post-trauma changes to the stress response system rather than the exposure to the stress itself. Thus, in order to understand this relationship, it is necessary to examine biochemical processes and changes that take place both during and after a traumatic event. The model proposes that during the traumatic event, as part of the “fight or flight” response, there is an increase in the level of endorphins in the brain (Kavushansky et al., 2013). Neuroimaging studies have suggested that, in addition to their well-known role in ameliorating physical pain, endorphins also serve to reduce distressing emotional responses (Liberzon et al., 2002; Zubieta et al., 2003). After trauma, the endorphin system habituates with a reduction in available opioid receptors (Liberzon et al., 2007; Pietrzak et al., 2014), producing a period of withdrawal and symptoms of emotional distress that may contribute to PTSD. Since alcohol can increase endorphin levels, PTSD patients will find that alcohol makes up for that lack of endorphin signaling and compensates for the endorphin withdrawal, leading to the use of alcohol as a way to self-medicate and avoid emotional distress (Volpicelli et al., 1999).

A similar hypothesis centers on the dysregulation of both the glutamatergic and GABAergic systems of PTSD patients, as revealed primarily by proton magnetic resonance spectroscopy studies. Glutamatergic abnormalities such as increases in glutamate in the temporal cortex and reductions in the anterior cingulate are thought to occur due to stress and trauma-induced overflow of glutamate that results in excitotoxicity and inflammatory processes, contributing to long-term problems with regulating stress responses in the central nervous system (Meyerhoff et al., 2014). In conjunction with the glutamate abnormalities seen in PTSD patients, there appears to be a reduction of cortical GABA levels in the parieto-occipital region (Meyerhoff et al., 2014; Rosso et al., 2014). Reduced GABA levels in this region correlate with the severity of PTSD symptoms, particularly insomnia (Meyerhoff et al., 2014). Interestingly, in a study of PTSD patients with alcohol use disorder, it was found that cortical glutamate and GABA levels in the parieto-occipital and temporal cortices were normalized when compared to PTSD patients without alcohol abuse disorder. GABA and glutamate levels in these regions were no longer correlated with PTSD symptom severity or sleep quality in the comorbid population, though the correlation was significant among PTSD patients without an alcohol use disorder (Pennington et al., 2014). These findings suggest that self-medication with alcohol among PTSD patients may help to stabilize glutamate and GABA levels, which could result at least initially in improved PTSD symptoms. However, the comorbid population also showed significant abnormalities suggesting structural and functional damage to the anterior cingulate cortex, all of which strongly correlated with increased PTSD symptom severity that would ultimately lead to worse outcomes in this group (Pennington et al., 2014).

## Overlapping Effects of Trauma and Drugs on Neuronal and Endocrine Substrates

As the previously described model indicates, some hypotheses invoke shared neural mechanisms to explain the frequent co-occurrence of PTSD and SUD, as opposed to just increased exposure to trauma and/or drugs of abuse. As will be reviewed in this section, several studies have found evidence of similarly dysregulated brain circuitry in both disorders, particularly in circuits involved in reward and cognitive processes. Therefore, it is possible that at the neural level both disorders can promote and potentiate one another by pushing the relevant circuitry toward a pathological state that favors both PTSD and SUD symptoms. Many of these mechanisms have been espoused as unidirectional, meaning either that PTSD predisposes toward SUD or vice versa, but in almost all cases the reverse causality would logically follow since both disorders are proposed to act on the same biological systems. This section will highlight some examples of biological systems thought to be affected similarly by both PTSD and SUD.

The first such example is the dopaminergic system, and more specifically dopaminergic projections from the ventral tegmental area in the midbrain to the striatum and prefrontal cortex. These are the mesolimbic and mesocortical systems respectively, and both are highly involved in regulating behavioral responses to rewarding stimuli (Schultz, 2002; Olsen, 2011). Not only is the mesolimbic system involved in mediating responses to natural rewards (e.g., eating, sexual behavior, and exercising), but it has also been proposed as the final common pathway for the rewarding properties of substances of abuse (Pierce and Kumaresan, 2006). These include psychostimulants (e.g., cocaine and amphetamine), ethanol, opiates, cannabinoids, and nicotine with all exerting pharmacological and physiological effects primarily by increasing dopamine transmission in the mesolimbic system either directly or indirectly (Pierce and Kumaresan, 2006). This reward-induced dopaminergic activity promotes motivated behaviors and links those behaviors to cues associated with the reward (Wyvell and Berridge, 2000; Sotak et al., 2005; Hamid et al., 2016).

Aversive and stressful experiences affect the dopaminergic reward pathway in ways that largely mimic the effects of addictive drugs. Both human and animal studies have shown that acute exposure to stress causes increased dopamine release in the nucleus accumbens (Abercrombie et al., 1989; Rougé-Pont et al., 1993; Kalivas and Duffy, 1995; Pruessner et al., 2004; Scott et al., 2006; Wood et al., 2007). Though the mechanisms are not entirely clear, animal studies have shown that this effect is at least partially mediated by activation of the hypothalamic-pituitary-adrenal (HPA) axis, components of which promote dopamine release (Piazza et al., 1996; Rougé-Pont et al., 1998). Stress enhances the effects of drug-related cues on the dopaminergic system, leading to increased cue-induced craving and reinstatement of drug self-administration (Liu and Weiss, 2002; Buffalari and See, 2009; Fox et al., 2014; Moran-Santa Maria et al., 2014). Clinical studies have also found that acute stress is strongly associated with an increased acute risk for relapse to drug use (Khantzian, 1985; Sinha, 2001).

Chronic exposure to alcohol and other drugs of abuse causes long-term changes in reward processing that are thought to promote a continued escalation of substance use. Even though positive hedonic feelings occur shortly after the drug intake, negative hedonic responses follow—especially after repeated exposures—due to alterations in the brain reward system and stress-related structures such as the extended amygdala, resulting in a withdrawal syndrome including dysphoria, irritability, anxiety, and other negative emotional states (Zhang and Schulteis, 2008; Leventhal et al., 2013; Su et al., 2017; Fleming et al., 2019). Some hypothesize that over time the desire to avoid the negative feelings associated with withdrawal becomes the primary motivational factor for compulsive drug-seeking behavior (Solomon and Corbit, 1974; Koob and Volkow, 2010). A key tenet of this opponent-process theory is that circuitry involved in producing the reinforcing effects of drugs of abuse eventually undergoes tolerance, resulting in long-term reductions in dopaminergic activity, an increased reward threshold, and a decreased desire to pursue natural rewarding stimuli (Volkow et al., 1997, 2007, 2014; Martinez et al., 2007). In what amounts to a more intricate version of the self-medication hypothesis, the experienced drug user is described as engaging in ever-increasing levels of drug use in an effort to overcome a chronic and deepening reward deficit.

A similar reward deficit is thought to be a central feature of PTSD, in which case it would be classified as a depressive-like anhedonia syndrome as described in Criterion D. It has been shown that PTSD patients are less likely to expend effort to gain access to a rewarding stimulus (Elman et al., 2005), and they report less reward expectancy and satisfaction if a reward is delivered (Hopper et al., 2008). Some of the underlying brain mechanisms are thought to include reduced activation of mesolimbic structures like the nucleus accumbens in response to positive gains as well as other regions crucial for reward processing including the medial prefrontal cortex (Sailer et al., 2008). PTSD patients have also been found to have an increased density of dopamine transporters in the striatum, which is thought to be a sign of greater dopamine turnover and perhaps reduced dopaminergic tone as dopamine is cleared more efficiently from the synapses (Hoexter et al., 2012). This is similar to the decreased striatal D2 receptor density observed during abstinence in patients with SUD that is thought to mediate withdrawal-related drug craving (Volkow et al., 1993, 2001). Thus, it is possible that alterations in reward circuits produced by PTSD and SUD complement and reinforce one another, resulting in anhedonic states that perpetuate both disorders.

In addition to the above-described anhedonia and overall decrease in dopaminergic activity, chronic drug use is also characterized by a sensitized, hyperdopaminergic response to drug-related cues, with associated increases in motor activity and motivated behaviors including drug self-administration (Kalivas and Stewart, 1991; Robinson and Berridge, 1993; Vezina, 2004). Though most of the original evidence for sensitization was derived from animal research (Robinson and Becker, 1986), behavioral and dopaminergic sensitization to drug cues has now been reported in several human studies as well (Boileau et al., 2006; O’Daly et al., 2011; Booij et al., 2016). This dopaminergic incentive-sensitization effect has often been portrayed as being in conflict with opponent-process, but incentive-sensitization can also be seen as a necessary complement to a theory that is attempting to explain both a general loss of interest in motivated behaviors and a simultaneous increase in one specific type of motivated behavior, namely substance use. The sensitization effect appears very specific to drug-related cues, because evidence of tolerance, rather than sensitization, is generally observed when such cues are absent (Leyton and Vezina, 2013). Sensitization is thought to occur because drugs of abuse directly or indirectly increase dopaminergic transmission in the nucleus accumbens (Hyman et al., 2006). Glutamatergic synapses that are involved in linking drug-related stimuli to drug-taking behavioral responses are active at the time of this dopamine release and are therefore strengthened every time the drug is used due to activation of relatively low-affinity dopamine type 1 receptors. In contrast, synapses representing non-drug related stimuli and actions are preferentially active in the presence of lower concentrations of dopamine that are more likely to activate high-affinity dopamine type 2 receptors, which will progressively weaken the synaptic strength in those circuits (Grace et al., 2007; Surmeier et al., 2007; Lovinger, 2010). Over time, the drug user’s thoughts and behaviors become increasingly funneled toward the drug and its related stimuli, at the expense of all other non-drug rewards regardless of how motivating they may have been in the past (Leyton and Vezina, 2014; Berridge and Robinson, 2016).

Repeated or prolonged exposure to stress can also recapitulate some of the core pathophysiology of SUD. Sensitization of the dopaminergic response to stress has been extensively documented with repeated stress exposure (Jordan et al., 1994; Tidey and Miczek, 1996; Naef et al., 2013), and the behavioral and neurochemical effects of repeated stress cross-sensitize with those of repeated drug exposure (Prasad et al., 1995; Piazza and Le Moal, 1996; Booij et al., 2016). Sensitization of the stress response has been documented in PTSD patients and is thought to be a core feature of the disorder (Dykman et al., 1997; Yehuda, 1997; Elzinga and Bremner, 2002). For example, when subjected to cognitive stress, male veterans suffering from PTSD have increased stress responses and adrenocorticotrophic hormone (ACTH) levels compared to controls, which reflects their higher distress level (de Kloet et al., 2012). It has also been reported that patients who are less responsive to PTSD therapy have salivary cortisol responses to trauma-related imagery that actually strengthens over the course of treatment rather than decreasing or remaining constant (Rauch et al., 2017). Animal studies suggest that, especially with repeated re-exposure to trauma-related cues, these conditioned stress responses can become progressively stronger and expand to other central neurochemical systems such as norepinephrine (Anisman and Sklar, 1979; Jedema et al., 2008; Chen et al., 2012) and serotonin (Adell et al., 1988; Zhang et al., 2014; Hasegawa et al., 2018).

Heightened stress responses lead to increased activity of norepinephrine neurons within the locus coeruleus due to stimulation by corticotropin-releasing factor (Curtis et al., 1997; Reyes et al., 2008). Activity in these norepinephrine neurons triggers a range of aversive and anxiety-like emotional responses (McCall et al., 2015; Hirschberg et al., 2017). Hyperactive norepinephrine signaling is thought to be a core feature of the pathophysiology of PTSD (Bremner et al., 1997; Yehuda et al., 1998; Geracioti et al., 2001; Pietrzak et al., 2013; Steuwe et al., 2014). It may also be involved in SUD, as human and animal studies have found elevations in both central and peripheral noradrenergic activity during all phases of substance use including acute intoxication, chronic use, withdrawal, and relapse (Hawley et al., 1981; Kovács et al., 2002; Patkar et al., 2003; Lanteri et al., 2008; Fitzgerald, 2013). This might suggest that blockade of excessive noradrenergic activity would be helpful for both SUD and PTSD. Indeed, the alpha-1 adrenergic antagonist prazosin has well-established efficacy for reducing PTSD nightmares (Raskind et al., 2003, 2007, 2013; Germain et al., 2012) and prazosin reduced drug self-administration in several animal studies (Walker et al., 2008; Greenwell et al., 2009; Rasmussen et al., 2009; Forget et al., 2010; Lê et al., 2011; Froehlich et al., 2015). Clinical trials of alpha-1 antagonists have also shown promise for the treatment of alcohol use disorder (Simpson et al., 2018; Wilcox et al., 2018). Despite these promising results for each individual disorder, so far prazosin has not been shown to improve outcomes for patients with comorbid PTSD and SUD (Petrakis et al., 2016; Verplaetse et al., 2019). Other noradrenergic agents have been tested with more mixed results for PTSD and SUD, but overall manipulation of the noradrenergic system remains a promising avenue for treatment of this difficult comorbidity.

The relationship between the serotonergic system and comorbid SUD and PTSD is less clear than for the other monoamines. The main evidence for serotonin playing an important role in the pathophysiology of PTSD comes from clinical responses to manipulations of the serotonergic system. Currently, selective serotonin reuptake inhibitors (SSRIs) are the only medications with an FDA approval for the treatment of PTSD (Brady et al., 2000; Davidson et al., 2001; Marshall et al., 2001). Acute reduction of serotoninergic activity using tryptophan depletion exacerbates PTSD symptoms (Corchs et al., 2015). However, administration of the serotonin agonist meta-chlorophenyl-piperamine also causes an acute exacerbation of PTSD symptoms (Southwick et al., 1995), and SSRIs have limited to no efficacy for many PTSD patients (Hertzberg et al., 2000; Zohar et al., 2002). There is some indirect evidence of serotonergic involvement in the development of SUD as well. As with dopamine and norepinephrine, animal studies have shown that most drugs of abuse acutely increase serotonin in both cortical and subcortical areas (Tao and Auerbach, 1995; Selim and Bradberry, 1996; Teneud et al., 1996; Singer et al., 2004; Pum et al., 2007), though cannabis is a notable exception that actually decreases serotonergic activity (Sano et al., 2008). The effects of chronic drug use on serotonin are also fairly consistent across classes, with chronic cocaine, alcohol and morphine causing long-term decreases (Parsons et al., 1995; McBride et al., 2004; Goeldner et al., 2011), but no clear effect of either chronic amphetamine or nicotine (Touiki et al., 2008; Barr et al., 2013). Selective serotonin reuptake inhibitors are generally not effective for SUDs (Kranzler et al., 1995; Lima et al., 2003; Hughes et al., 2014), though some studies indicate that antidepressants for alcohol use disorder may improve outcomes for some patients and worsen outcomes for others depending on family history and pattern of alcohol use (Pettinati et al., 2000; Chick et al., 2004; Kranzler et al., 2012). Clinical trials of SSRIs for comorbid SUD and PTSD have thus far not been promising (Brady et al., 2005; Petrakis et al., 2012).

## Personality/Behavioral Traits

Because of the often complex interactions between relevant genetic and environmental factors, it can be difficult to recognize individual factors that affect vulnerability to PTSD and SUD. Behavioral endophenotypes are more closely related to the abnormalities that characterize these disorders and have the potential to integrate many different underlying genetic and environmental factors, in effect providing a valuable summary of data that might otherwise be prohibitively difficult or impossible to get (Gottesman and Gould, 2003). One such endophenotype that has been consistently associated with both PTSD and SUD is impulsivity (Weiss et al., 2013; James et al., 2014; Walker et al., 2018; Grubbs and Chapman, 2019). Impulsivity is a multifaceted concept in research but can be broadly defined as a tendency to engage in risky, premature, or situationally inappropriate actions that are characterized by a lack of planning or forethought (Robbins et al., 2012; Jentsch et al., 2014; Dalley and Robbins, 2017). As has been found in patients with PTSD and SUD, impulsivity is associated with lower dopaminergic activity in the NAc at baseline and in response to neutral cues, but exaggerated striatal responses to more salient cues (Forbes et al., 2009; Hahn et al., 2009; Lee et al., 2009; Colzato et al., 2010; O’Sullivan et al., 2011; Reeves et al., 2012). Impulsivity is also thought to result from impaired prefrontal cortical control over motivationally relevant signals from the NAc and other subcortical structures (Rolls et al., 1994; Aron et al., 2004; Schmaal et al., 2012; Davis et al., 2013). This same pattern of prefrontal hypoactivity that is insufficient to restrain subcortical impulses has been identified in functional neuroanatomical studies as a key etiologic factor for both SUD and PTSD in preclinical studies (Peters et al., 2009; Goode and Maren, 2019).

Another related behavioral trait is “cue reactivity,” or a tendency towards exaggerated neuronal, emotional and motivational responses to stimuli that have been associated with emotionally salient events. The relevance of cue reactivity to PTSD is clear because excessive fear responses triggered by trauma cues is a core diagnostic feature of PTSD, and the intensity of trauma cue reactions correlates well with PTSD symptom severity (Shin et al., 2004; Rabellino et al., 2016; Rauch et al., 2017). Cue reactivity to drug-related stimuli also predicts relapse in patients with SUD (Rohsenow et al., 1990; Carter and Tiffany, 1999; Janes et al., 2010). Reactions to drug- and trauma-cues seem to intensify and reinforce one another among patients with PTSD and SUD. For example, one study measured visual, physiological, and behavioral responses of patients with comorbid PTSD and cocaine use disorder to cues associated with both their trauma and preferred drug. Compared to those of patients with a single diagnosis of cocaine use disorder and age- and gender-matched controls, patients with dual-diagnosis had excessive cue-reactivity to both the trauma- and drug-related visual cues (Sokhadze et al., 2008). Trauma cues elicit higher levels of distress and negative emotion in patients with comorbid PTSD and SUD when they are accompanied by drug-related imagery (Coffey et al., 2002). Patients with comorbid PTSD and SUD also show more intense drug cue reactivity, including increased cravings to use drugs, when exposed to personalized trauma cues (Coffey et al., 2010; Tull et al., 2011; Read et al., 2017). Both PTSD and SUD patients have a tendency to act impulsively in response to emotionally charged stimuli, a trait that is known as “emotional urgency” (Whiteside and Lynam, 2001; Cyders and Smith, 2008), and this tendency correlates with symptom severity and functional impairment (Ehring and Quack, 2010; Smith and Cyders, 2016). These findings suggest that symptoms of emotional urgency, impulsivity and cue reactivity are interrelated and may cross-sensitize in PTSD and SUD, thereby exacerbating the severity of both illnesses.

In addition to its role in the pathophysiology of PTSD and SUD, cue reactivity may also be a pre-existing behavioral trait that predisposes individuals to develop these disorders. This possibility has mainly been explored in preclinical studies by comparing animals that exhibit individual variation in their reactivity to conditioned cues. An example of this is “sign-trackers” (STs) and “goal-trackers” (GTs) which can be identified using a Pavlovian conditioned approach procedure. When a food reward is paired with a localizable cue such as a retractable lever, STs approach and are attracted to the cue itself, whereas GTs direct their attention away from the cue and towards the location of impending reward delivery (Flagel et al., 2009; Tomie and Morrow, 2018). Sign-tracking is thought to indicate vulnerability to SUD because STs show increased psychomotor sensitization to cocaine (Flagel et al., 2008), have higher preference for cocaine over food (Tunstall and Kearns, 2015) and show increased cue-induced reinstatement of nicotine (Versaggi et al., 2016) and cocaine (Saunders and Robinson, 2011). In addition, it has been shown that STs, identified by the high levels of incentive salience they attribute to reward-related cues, also show elevated fear responses to a tone that has been paired to a foot-shock (Morrow et al., 2011). This indicates that the sign-tracking trait may represent a more general tendency to attribute excessive motivational salience to cues paired with biologically relevant events, regardless of emotional valence. Sign-tracking may therefore also be a risk factor for PTSD, as suggested by evidence that repeated exposure of STs to aversive stimuli results in a fear response that increases over time, instead of decreasing or remaining stable as is the case for GTs (Morrow et al., 2015). Sign-tracking has not yet been studied in human PTSD patients, but as described in the previous paragraph the related trait of cue-reactivity is elevated in subjects with PTSD.

It is important to note that the exaggerated emotional and motivational cue reactivity of STs is specifically tied to discrete, localizable cues. There are no differences between STs and GTs in learning instrumental tasks, so general associative learning and memory processes appear to be intact in both phenotypes (Ahrens et al., 2016; Fitzpatrick et al., 2019). However, STs show lower levels of contextual fear than GTs, as well as decreased context-induced reinstatement of drug self-administration (Morrow et al., 2011; Saunders et al., 2014). Thus, STs tend to react strongly to conditioned cues regardless of the circumstances under which they are encountered, whereas GTs use contextual cues to modulate their conditioned emotional responses (Pitchers et al., 2017). Patients with PTSD show exactly these kinds of deficits; their learned fear responses are insensitive to contextual shifts, safety signals, or other indicators of whether the present circumstances are “safe” or “unsafe” (Maren et al., 2013; Garfinkel et al., 2014; Liberzon and Abelson, 2016). For example, extinction learning is impaired in PTSD patients such that they show relatively high levels of fear in “safe” contexts (Milad et al., 2009; Wicking et al., 2016), but in renewal tests, PTSD patients also fail to show increased fear in the “unsafe” context (Garfinkel et al., 2014). Again this does not appear to be due to a general learning deficit, as PTSD patients do not differ from controls in explicitly or “cognitively” differentiating “safe” and “unsafe” contexts (Steiger et al., 2015). Rather, the difficulty appears to be specifically in using contextual information to modulate the conditioned emotional response to cues. According to this conceptualization, the problem with fear in PTSD is not that the fear response is too strong. After all, intense fear in life-threatening situations is perfectly normal. It is the expression of fear in inappropriate circumstances that makes these reactions pathological. Drug use is also a normal human behavior, as evidenced by lifetime use estimates in the United States of 48% for illicit drugs, 63% for tobacco products, and 80% for alcohol (Substance Abuse and Mental Health Services Administration, 2018). However over the course of addiction substance use occurs in increasingly inappropriate contexts, such that it comes to interfere with work, relationships, and other important responsibilities. Though there is substantial evidence that physiological and behavioral consequences of drug use can be highly context-dependent (Crombag et al., 2001; Badiani, 2013), there is also evidence of decreased contextual modulation of responses to drug cues among SUD patients as compared to subjects who used drugs but do not have SUD (Garland et al., 2018). Thus, a failure to use contextual information in order to appropriately modify conditioned responses to emotionally salient cues may be a common feature of both PTSD and SUD.

## Shared Genetic Factors

The high rates of comorbidity between neuropsychiatric disorders have suggested that many of them might share common genetic risk factors. It has been proposed that genetic overlap may help to explain the frequent co-occurrence of externalizing disorders like SUD with internalizing disorders such as PTSD, generalized anxiety disorder, and major depressive disorder (Kendler et al., 2003). Twin studies have provided some evidence for genetic commonalities between PTSD and SUD (Xian et al., 2000; McLeod et al., 2001; Koenen et al., 2003; Wolf et al., 2010). For example, in the year 2000, Xian et al. (2000), conducted a study on 3,304 male-male twin pairs from the Vietnam Era Twin Registry (VETR) to examine the genetic overlap of PTSD with alcohol dependence (AD) and drug dependence (DD). According to their study, the risk for PTSD was due to 15.3% common genetics with AD and DD, while risk for AD was accounted for by 55.7% common genetics with PTSD and DD (Xian et al., 2000). Similarly to that found in males, a study of 3,768 female-female twin pairs found that trauma exposure and PTSD had a significant genetic correlation with AD accounting for 28% of its genetic variance (Sartor et al., 2011). Interestingly, another study with twins registered in the VETR focused on how anxiety and mood disorders loaded on externalizing and internalizing factors. They showed that PTSD was unique in the sense that it loaded on both externalizing and internalizing factors while none of the other anxiety/mood disorders loaded on externalizing factors (Wolf et al., 2010). They concluded that the high comorbidity between these internalizing and externalizing disorders can be attributed to genetic factors that predispose to both types of disorders.

Several studies have also focused on specific genetic variants. Of particular interest has been the D2 dopamine receptor (D2DR) *Taq*I A1 allele which has been previously implicated in alcohol (Neiswanger et al., 1995; Lawford et al., 1997; Dahlgren et al., 2011) and DD (Noble et al., 1993; Comings et al., 1996; Lawford et al., 2000; Li et al., 2019). A study of military veterans found that this polymorphism was more frequent in PTSD patients, but only in those who were also harmful drinkers. PTSD patients that were not harmful drinkers did not differ in their A1 allele frequency when compared to controls with a low-risk level of alcohol consumption. In addition, they found that PTSD patients with the A1 allele drank more than twice the amount of alcohol compared to PTSD patients lacking the allele (Young, 2002). In humans, the D2DR A1 allele has been previously linked to reduced density of D2 receptors in the striatum (Noble et al., 1993; Pohjalainen et al., 1998; Jönsson et al., 1999), which is thought to contribute to a hypodopaminergic state and the reward deficiency syndrome associated with SUD and PTSD (Blum et al., 2000, 2012; Elman et al., 2005, 2018; Hopper et al., 2008). In preclinical models, reduced baseline levels of striatal D2 receptors has served as a predictor of an increased rate of cocaine self-administration in both rats (Dalley et al., 2007) and non-human primates (Nader et al., 2006). Interestingly, in rats, this reduced D2 receptor levels in the nucleus accumbens are correlated with a trait of impulsivity as measured by the five-choice serial reaction time task (Dalley et al., 2007). As described in the previous section, impulsivity is thought to be a behavioral endophenotype associated with increased vulnerability to both SUD and PTSD. Selectively bred alcohol-preferring P rats also show reduced D2 receptor levels in the nucleus accumbens (McBride et al., 1993), and upregulation of the D2 receptor in this region decreases both ethanol preference and intake in these rats (Thanos et al., 2001). Similarly, in rats that have been trained to self-administer cocaine, treatment with a D2R vector to increase expression in the nucleus accumbens attenuated the amount of cocaine infusions and lever presses for cocaine, an effect that lasted 6 days (Thanos et al., 2008). In mice, exposure to chronic mild stress induces increased ethanol intake and preference in heterozygous *Drd2*+/− mice compared to wild-type (Delis et al., 2013). *Drd2*+/− mice exposed to chronic mild stress also show increased immobility during the forced swim test, but this is reversed by ethanol intake, supporting the link between SUD and stress regulation (Delis et al., 2013). In preclinical models of PTSD-like symptoms, modulation of the D2 receptor has proven promising in attenuating negative symptoms. For example, in rats subjected to a single prolonged stress (SPS) procedure that mimics psychological trauma, administration of the D2 partial agonist aripiprazole corrects the context- and cue-induced extinction retrieval impairment produced by SPS (Lin et al., 2019). In a similar manner, the D2/D3 agonist rotigotine reduced exaggerated conditioned auditory fear responses and also reduced immobility in the forced swim test in mice that had been subjected to SPS (Malikowska-Racia et al., 2019).

Another gene of interest is the 5-HTTLPR polymorphism of the serotonin transporter, which has been associated with stress reactivity (Gunthert et al., 2007; Gotlib et al., 2008; Miller et al., 2013; Alexander et al., 2014). A study of environmental and genetic factors in children found that the early use of alcohol could be predicted by an interaction between history of maltreatment and the 5-HTTLPR polymorphism (Kaufman et al., 2007). Another study conducted in adults found that reported experiences of childhood adversity and adult traumatic events predicted PTSD. Again, the 5-HTTLPR polymorphism did not predict PTSD alone, but it did increase risk when combined with childhood and adult traumas (Xie et al., 2009, 2012). This polymorphism is thought to reduce expression levels of the serotonin transporter by impairing the transcriptional efficiency of the gene promoter (Lesch et al., 1996). Thus, translational value has been the SERT knockout and knockdown rodent models. Studies have shown that these mice do not differ in their baseline levels of stress hormones, but in response to a physical stressor, SERT+/− and SERT−/− mice show exaggerated responses of plasma ACTH levels (Murphy et al., 2001) as well as increased plasma epinephrine in SERT−/− (Tjurmina et al., 2002) suggesting alterations in the HPA axis stress-induced response. In addition, exposure to a predator odor induces long-lasting anxiogenic effects in SERT−/− mice as measured by increased anxiety-like behaviors in the plus maze and light/dark box test when compared to wild-type, which may be relevant as a model of increased vulnerability to PTSD (Adamec et al., 2006). SERT−/− mice also show enhanced cocaine-conditioned place preference when compared to wild-type mice (Sora et al., 1998), and SERT−/− rats also show enhanced cocaine-conditioned place preference in addition to an increased psychomotor response to cocaine and intravenous self-administration (Homberg et al., 2008). Altogether, both clinical and preclinical data suggest that alterations in the expression of the serotonin transporter can affect both stress responsivity and the rewarding properties of addictive substances which can influence vulnerability to both SUD and PTSD.

## Early Life Stress

The impact of early life stress (ELS) on predisposition to neuropsychiatric disorders has been widely studied, particularly with regard to SUD and PTSD (Bremner et al., 1993; Heim and Nemeroff, 2002; Enoch, 2011; Rodrigues et al., 2011; Syed and Nemeroff, 2017; Walters and Kosten, 2019). The HPA-axis plays a central role in mediating stress responses and restoring basal states following a stressor (Habib et al., 2001; Smith and Vale, 2006; Lightman and Conway-Campbell, 2010). Early life stressors, which include neglect as well as physical, emotional, and sexual abuse (Bernstein et al., 1994), can have long-lasting effects on the HPA-axis and manifest as maladaptive behaviors later in adulthood due to both cognitive and emotional impairments. The developing brain is particularly sensitive to external influences, which can alter gene expression, neurochemical balance, neuronal maturation, and synaptic function both basally and in response to stress (Weinstock, 2005, 2008; Glover et al., 2010; van Bodegom et al., 2017; Matthews and McGowan, 2019).

As previously described, disruptions of the stress response due to dysregulation of the HPA-axis as well as altered reward processing due to imbalances in mesolimbic activity can increase susceptibility to both SUD (Piazza et al., 1996; Volkow et al., 1997, 2014; Rougé-Pont et al., 1998; Martinez et al., 2007) and PTSD (Yehuda, 1997; Sailer et al., 2008). In rats, ELS is associated with anxiety-like behaviors such as impaired fear extinction (Judo et al., 2010; Bingham et al., 2013; Wilson et al., 2013), and enhanced psychomotor responses to alcohol (Kawakami et al., 2007), opiates (Kalinichev et al., 2002), amphetamine (Henry et al., 1995; Kehoe et al., 1996; Brake et al., 2004), and cocaine (Kehoe and Boylan, 1992; Brake et al., 2004; Thomas et al., 2009; Anier et al., 2014) as well as enhanced acquisition of cocaine (Kosten et al., 2000; Flagel et al., 2003), methamphetamine (Lewis et al., 2013), and alcohol (Gondré-Lewis et al., 2016) self-administration. In addition, human data shows that maltreated children report alcohol use seven times higher than that of control children as well as an earlier age of drinking initiation, which are predictors of future AD (Kaufman et al., 2007). Furthermore, later in adulthood, the level of substance use positively correlates with both PTSD symptoms and the level of sexual, physical, and emotional childhood trauma (Khoury et al., 2010).

In rats, many studies have shown that pre- and neo-natal stress (e.g., restraint, hypoxia, foot-shocks, etc. to the mother or maternal separation, social deprivation, etc. to the pups) can alter both basal and stressed-induced CRH, ACTH, and corticosterone levels through activation of the HPA-axis, changes which persist into adulthood. As is also the case with the human literature, there has been variability in the results of animal studies due to influences of sex, developmental stage during the stress exposure, the nature of the stressor and its duration, and the age at testing (Weinstock, 2008; van Bodegom et al., 2017). Nonetheless, most studies have been consistent with showing some type of alteration in HPA reactivity. These changes are accompanied by adaptations in the limbic and cortical system, such as increases in the expression of CRHR1 in regions like the PVN (Bravo et al., 2011; Fan et al., 2013; Wang et al., 2013), the amygdala (Bravo et al., 2011; Brunton et al., 2011), the hippocampus (O’Malley et al., 2011), and the prefrontal cortex (Vázquez et al., 2003; O’Malley et al., 2011), which is thought to be crucial for initiation of the stress response (Bale and Vale, 2004; Henckens et al., 2016). In addition, decreased expression of the glucocorticoid receptor in the hippocampus (Henry et al., 1994; Levitt et al., 1996; Green et al., 2011; Bingham et al., 2013) and prefrontal cortex (Green et al., 2011; Bingham et al., 2013) is thought to disrupt the ability of the system to respond appropriately to feedback loops and control stress responses (Jacobson and Sapolsky, 1991; de Kloet et al., 1993; Herman and Cullinan, 1997; Mizoguchi et al., 2003; Herman et al., 2012). ELS results in exaggerated responses to psychological stressors as seen in adult animals exposed to pre- and post-natal stress (Engelmann et al., 1996; Vallée et al., 1997; Tazumi et al., 2005; Brunton and Russell, 2010). As previously described, PTSD patients show dysregulation of the HPA-axis (de Kloet et al., 2006; Dunlop and Wong, 2019). Although basal levels of cortisol seem to be decreased in many studies (Mason et al., 1986; Yehuda et al., 1990; Yehuda and Seckl, 2011), the stress response to trauma-related cues is exaggerated and perpetuated due to the inability of the system to restore homeostasis (Yehuda, 1997; Elzinga and Bremner, 2002). Importantly for the relationship between ELS, PTSD and SUD, the interaction between the stress and dopaminergic systems seems to be carefully coordinated, suggesting reciprocal modulation between the two systems (Härfstrand et al., 1986; Piazza and Le Moal, 1996; Piazza et al., 1996; Marinelli and Piazza, 2002; Rougé-Pont et al., 1998). In the mesolimbic circuit, particularly within the VTA-NAc projection, ELS causes long-term changes in dopaminergic activity. Both animal and human studies have reported an increase in stress-induced dopamine release during adulthood (Hall et al., 1999; Brake et al., 2004; Pruessner et al., 2004; Yorgason et al., 2013), and animal studies have shown long-lasting changes in dopamine receptors including decreased NAc D2R expression in prenatally stressed rats treated with nicotine (Said et al., 2015) as well as in rats subjected to maternal separation (Majcher-Maślanka et al., 2017). As previously mentioned, decreased density of the D2R in the NAc is thought to be an important contributor to drug craving (Volkow et al., 1993, 2001).

Another target of ELS is the hippocampus, which expresses a high density of glucocorticoid receptors (Jacobson and Sapolsky, 1991; Maras and Baram, 2012). ELS can impair hippocampal development by degrading its structure and function. The impairments in learning and memory associated with ELS are thought to be mainly mediated by significant reductions in hippocampal volume and synaptic activity. ELS causes decreased neurogenesis and cell proliferation, reductions in spine density, dendritic atrophy, and disruption of synaptic pruning (Lemaire et al., 2000; Andersen and Teicher, 2004; Brunson et al., 2005; Ivy et al., 2010; Oomen et al., 2010; Hulshof et al., 2011). These changes can directly impact synaptic plasticity in the form of impaired long-term potentiation and facilitated long-term depression (Brunson et al., 2005; Yang et al., 2006; Ivy et al., 2010). For example, in rats subjected to contextual fear conditioning, ELS causes synaptic inhibition between the hippocampus and the medial prefrontal cortex in response to the extinction trials, and this is accompanied by persistent freezing due to impaired extinction retrieval of the fear memory (Judo et al., 2010), a feature of PTSD in humans (Maren et al., 2013; Garfinkel et al., 2014). Decreased hippocampal volume as a result of ELS has been reported in both animal and human models (Uno et al., 1994; Andersen and Teicher, 2004; Humphreys et al., 2019) and this is consistent with significant hippocampal volume reductions in combat- and childhood-related PTSD (Bremner et al., 1995, 1997; Karl et al., 2006; Wang et al., 2010) that worsens over the duration of the disorder (Felmingham et al., 2009; Chao et al., 2014). These findings are not confined to PTSD; reduced hippocampal function has also been identified as a feature of AD (Agartz et al., 1999; Beresford et al., 2006), cannabis dependence (Chye et al., 2019), and methamphetamine psychosis (Orikabe et al., 2011).

The prefrontal cortex is another important player in the inhibition of stress responses and regulation of cognitive and emotional processing (Diorio et al., 1993; Ochsner and Gross, 2005; Herman et al., 2012). ELS has been linked to reduced prefrontal cortical volume and function in adulthood, most likely due to the permanent impact these neurophysiological changes can have on the region while it is still undergoing development (Chocyk et al., 2013). The prefrontal cortex normally regulates subcortical processing of both appetitive and aversive cues. Both decreased volume and hypoactivity of the prefrontal cortex have been linked to anxiety and addiction disorders. In rats, postnatal stress causes a volumetric reduction of the prefrontal cortex (Sarabdjitsingh et al., 2017) and this is consistent with human data showing that childhood emotional maltreatment reduces the volume of the medial prefrontal cortex (van Harmelen et al., 2010) and results in hypoactivity within this region in adulthood (van Harmelen et al., 2014). Patients with PTSD also show hypoactivity of the ventromedial prefrontal cortex (Hayes et al., 2012) and significant thinning (Geuze et al., 2008) that correlates with the severity of their PTSD symptoms (Wrocklage et al., 2017). Reduced prefrontal cortical volumes have also been observed in SUD patients (Liu et al., 1998; Fein et al., 2002; Schlaepfer et al., 2006; Tanabe et al., 2009; Becker et al., 2015), along with a range of deficits in executive control indicating functional impairment of the prefrontal cortex (Goldstein et al., 2004; Goldstein and Volkow, 2011; Ramey and Regier, 2019). In addition, ELS has been shown to blunt the increase in prefrontal D2R expression that normally occurs during adolescence (Brenhouse et al., 2013). This may suggest a link between ELS and subsequent SUD risk since rats exposed to a long access self-administration paradigm show a decrease in D2R mRNA and protein expression in the orbital prefrontal cortex in conjunction with impaired sustained attention (Briand et al., 2008).

Similar to the glutamatergic synaptic overflow reported in PTSD patients and reduced cortical levels of GABA, ELS by maternal separation in mice leads to increased basal levels of glutamate release in the somatosensory cortex as well as increased glutamate release after a nociceptive stressor. Maternally separated mice seem to show an inability to restore glutamatergic homeostasis which may, in turn, affect glucocorticoid secretion in response to stressors (Toya et al., 2014). In addition, in the hippocampus, maternal separation in rats seems to also affect the glutamatergic/GABAergic, excitatory/inhibitory ratio by affecting the expression of proteins responsible for the cycling of these neurotransmitters. This includes upregulation of EAAT1 and EAAT2, which is in accordance with a homeostatic response to increased synaptic glutamatergic levels, as well as downregulation of VGAT and GAD63 which may suggest reduced GABA levels (Martisova et al., 2012). These alterations are thought to be mainly mediated by stress-induced corticosterone release, as chronic treatment with corticosterone recapitulated most of the effects seen in maternally separated rats (Martisova et al., 2012).

Overall, these data suggest that exposure to ELS induces long-lasting effects in the HPA-axis and stress response system that in turn influence the activity of key neurotransmitters like dopamine, glutamate and GABA, and impair proper development and function of structures like the hippocampus and prefrontal cortex. As was described, many of the features that characterize ELS overlap with those previously linked with PTSD and SUD and may help explain why ELS is such a powerful risk factor for developing these disorders in adulthood.

## Conclusion

Based on the data from patients and animal models, many neurobiological and biochemical factors could be implicated in the development of PTSD and SUD comorbidity. So far, the exact neurobiological underpinnings for this comorbidity remain unknown. There are some putative explanations, e.g., self-medication, that do not require any direct neurobiological relationship between PTSD and SUD. However, based on multiple overlapping psychological and physiological effects of trauma and abuse substances, there are likely several neurobiological mechanisms whereby the development of one disorder can impact the development of the other. Much of the evidence reviewed in this article suggest that chronic exposure to either stress or drugs of abuse can push the mesolimbic motivational system into a state that is poised to react to salient stimuli with a surge of emotional and motivational activity that may be difficult to restrain and result in a multitude pathological behaviors such as those seen in both PTSD and SUD. The study of individual differences in vulnerability to develop these disorders may provide further insight into the surprising prevalence of comorbid PTSD and SUD, especially since both disorders represent a relatively uncommon reaction to the nearly ubiquitous experiences of trauma and substance use. A better understanding of the neurobiology and basic psychological processes that can predispose toward both PTSD and SUD would assist in the rational design of more effective treatment strategies aimed specifically at patients vulnerable to comorbid psychiatric disorders.

## Author Contributions

Both CM-R and JM contributed equally to the initial draft and subsequent revisions of this article.

## Conflict of Interest

The authors declare that the research was conducted in the absence of any commercial or financial relationships that could be construed as a potential conflict of interest.
